# Classification of optic disc shape in glaucoma using machine learning based on quantified ocular parameters

**DOI:** 10.1371/journal.pone.0190012

**Published:** 2017-12-19

**Authors:** Kazuko Omodaka, Guangzhou An, Satoru Tsuda, Yukihiro Shiga, Naoko Takada, Tsutomu Kikawa, Hidetoshi Takahashi, Hideo Yokota, Masahiro Akiba, Toru Nakazawa

**Affiliations:** 1 Department of Ophthalmology, Graduate School of Medicine, Tohoku University Graduate School of Medicine, Sendai, Japan; 2 Department of Ophthalmic Imaging and Information Analytics, Tohoku University Graduate School of Medicine, Sendai, Japan; 3 R&D Division, TOPCON Corporation, Tokyo, Japan; 4 Cloud-Based Eye Disease Diagnosis Joint Research Team, RIKEN, Wako, Japan; 5 Division of Ophthalmology, Tohoku Medical and Pharmaceutical University, Department of Medicine, Sendai, Japan; 6 Image Processing Research Team, RIKEN, Wako, Japan; 7 Department of Retinal Disease Control, Ophthalmology, Tohoku University Graduate School of Medicine, Sendai, Japan; 8 Department of Advanced Ophthalmic Medicine, Tohoku University Graduate School of Medicine, Sendai, Japan; Massachusetts Eye & Ear Infirmary, Harvard Medical School, UNITED STATES

## Abstract

**Purpose:**

This study aimed to develop a machine learning-based algorithm for objective classification of the optic disc in patients with open-angle glaucoma (OAG), using quantitative parameters obtained from ophthalmic examination instruments.

**Methods:**

This study enrolled 163 eyes of 105 OAG patients (age: 62.3 ± 12.6, mean deviation of Humphrey field analyzer: -8.9 ± 7.5 dB). The eyes were classified into Nicolela’s 4 optic disc types by 3 glaucoma specialists. Randomly, 114 eyes were selected for training data and 49 for test data. A neural network (NN) was trained with the training data and evaluated with the test data. We used 91 types of quantitative data, including 7 patient background characteristics, 48 quantified OCT (swept-source OCT; DRI OCT Atlantis, Topcon) values, including optic disc topography and circumpapillary retinal nerve fiber layer thickness (cpRNFLT), and 36 blood flow parameters from laser speckle flowgraphy, to build the machine learning classification model. To extract the important features among 91 parameters, minimum redundancy maximum relevance and a genetic feature selection were used.

**Results:**

The validated accuracy against test data for the NN was 87.8% (Cohen’s Kappa = 0.83). The important features in the NN were horizontal disc angle, spherical equivalent, cup area, age, 6-sector superotemporal cpRNFLT, average cup depth, average nasal rim disc ratio, maximum cup depth, and superior-quadrant cpRNFLT.

**Conclusion:**

The proposed machine learning system has proved to be good identifiers for different disc types with high accuracy. Additionally, the calculated confidence levels reported here should be very helpful for OAG care.

## Introduction

Glaucoma is an optic neuropathy in which visual disturbance corresponds to optic disc cupping and optic nerve fiber degeneration [1]. Lowering intraocular pressure (IOP) is an effective, evidence-based treatment for open-angle glaucoma (OAG) [2,3], but meta-analysis has shown that non-IOP risk factors also contribute to progression [4], and glaucoma is now regarded as multifactorial [5]. Therefore, ophthalmologists must consider IOP-independent factors and varying pathophysiologies in glaucoma patients, and adjust treatments strategies accordingly, to most effectively preserve quality of life.

Nicolela et al. described characteristic inter-individual variations in optic disc morphology, and classified the glaucomatous disc into 4 types [6]. This revealed disc-dependent variations in age, rate of spasm, arteriosclerosis, and myopia in patients. Follow-up investigations showed that disc type classification is a useful addition to the management of OAG [7–10]. However, classification of the optic disc can sometimes be difficult because it relies on subjective assessment methods. Thus, we previously attempted to develop new, objective, and more accurate methods of classifying the optic disc, based on stereophotography [11] and optical coherence tomography (OCT) [12].

Recently, machine learning technology has seen dramatic progress, and has enabled the development of new algorithms to diagnose age-related macular disease and glaucoma [13–15]. Thus, in this report, we used a variety of parameters to set up a machine-learning-based system for objective optic disc classification. We then investigated the accuracy of this method in training and testing groups randomly selected from among OAG patients at our clinic. Our results suggest that our method is highly reproducible, and that it might contribute not only to daily glaucoma care, but also to ophthalmological research by enabling big-data analysis in clinical trials of new disc-type-specific treatments for glaucoma.

## Material and methods

### Subjects

This study included 163 eyes of 105 OAG patients with a glaucomatous visual field meeting the Anderson-Patella classification criteria [16]. All patients underwent testing with the Humphrey field analyzer (HFA, SITA standard 24–2, Carl Zeiss Meditec) with only reliable and repeatable results being included. Patients were excluded if they had a spherical equivalent (SE) refractive error of < -8.00 diopters, ocular disease other than OAG, systemic disease affecting the visual field, or cataract progression. This study adhered to the tenets of the Declaration of Helsinki, and the protocols were approved by the Clinical Research Ethics Committee of the Tohoku University Graduate School of Medicine (study 2014-1-805). Participants provided their written informed consent to participate in this study. The ethics committees approved this consent procedure. In this study, minors were not included. The patients were classified into Nicolela’s 4 types: focal ischemic (FI), generalized enlargement (GE), myopic (MY), and senile sclerotic (SS) with a method we have previously described [11,12]. The distinctive characteristics of each disc type include rim notching in FI, a diffusely enlarged, rounded cup in GE, a tilted disc and temporal crescent peripapillary atrophy (PPA) in MY, and shallow cupping and haloing in SS. Three glaucoma specialists performed the classification (TN, KO, and ST). Cases were excluded when classification was not consistent between all three graders. Demographic data are listed in Table 1 and assignment data are listed in Table 2. There were no significant differences in background between training and test data (t-test).

**Table 1:** Demographic data.

	All	FI^a^	GE^b^	MY^c^	SS^d^
	n = 163	n = 26	n = 50	n = 55	n = 32
**Male / Female**	85 / 78	9 / 17	31 / 19	27 / 28	18 / 14
**Age (years)**	62.3 ± 12.6	63.0 ± 11.9	65.7 ± 10.0	53.6 ± 11.6	71.5 ± 9.1
**MD**^e^ **(dB)**	-8.9 ± 7.5	-5.2 ± 5.8	-12.9 ± 7.7	-7.8 ± 7.1	-7.8 ± 6.0
**SE**^f^ **(D**^g^**)**	-2.5 ± 2.9	-1.2 ± 1.7	-0.5 ± 1.5	-5.6 ± 2.2	-1.5 ± 2.1
**IOP**^h^ **(mmHg)**	13.3 ± 3.5	13.6 ± 3.4	13.3 ± 2.9	13.4 ± 2.9	13.3 ± 2.9

^a^FI: focal ischemic,

^b^GE: generalized enlargement,

^c^MY: myopic,

^d^SS: senile sclerotic,

^e^MD: Humphrey-field analyzer (HFA)-measured mean deviation,

^f^SE: spherical equivalent,

^g^D: diopters,

^h^IOP: intraocular pressure.

Data are presented as the mean ± standard deviation.

**Table 2:** Assignment data.

	Training data				Test data				*P* value
	n = 114				n = 49				
	FI^a^	GE^b^	MY^c^	SS^d^	FI	GE	MY	SS	
	18	35	39	22	8	15	16	10	
**Male / Female**	58 / 56				27 / 22				1.000
**Age (years)**	62.8 ± 12.5				61.1 ± 13.0				0.432
**MD**^e^ **(dB)**	-9.6 ± 7.6				-7.4 ± 7.0				0.085
**SE**^f^ **(D**^g^**)**	-2.6 ± 2.9				-2.4 ± 3.1				0.693
**IOP**^h^ **(mmHg)**	13.3 ± 2.6				13.1 ± 2.4				0.646

^a^FI: focal ischemic,

^b^GE: generalized enlargement,

^c^MY: myopic,

^d^SS: senile sclerotic,

^e^MD: Humphrey-field analyzer (HFA)-measured mean deviation,

^f^SE: spherical equivalent,

^g^D: diopters,

^h^IOP: intraocular pressure.

Data are presented as the mean ± standard deviation. Differences were considered significant at p < 0.05.

### Measurement of clinical variables

**Fig 1:**
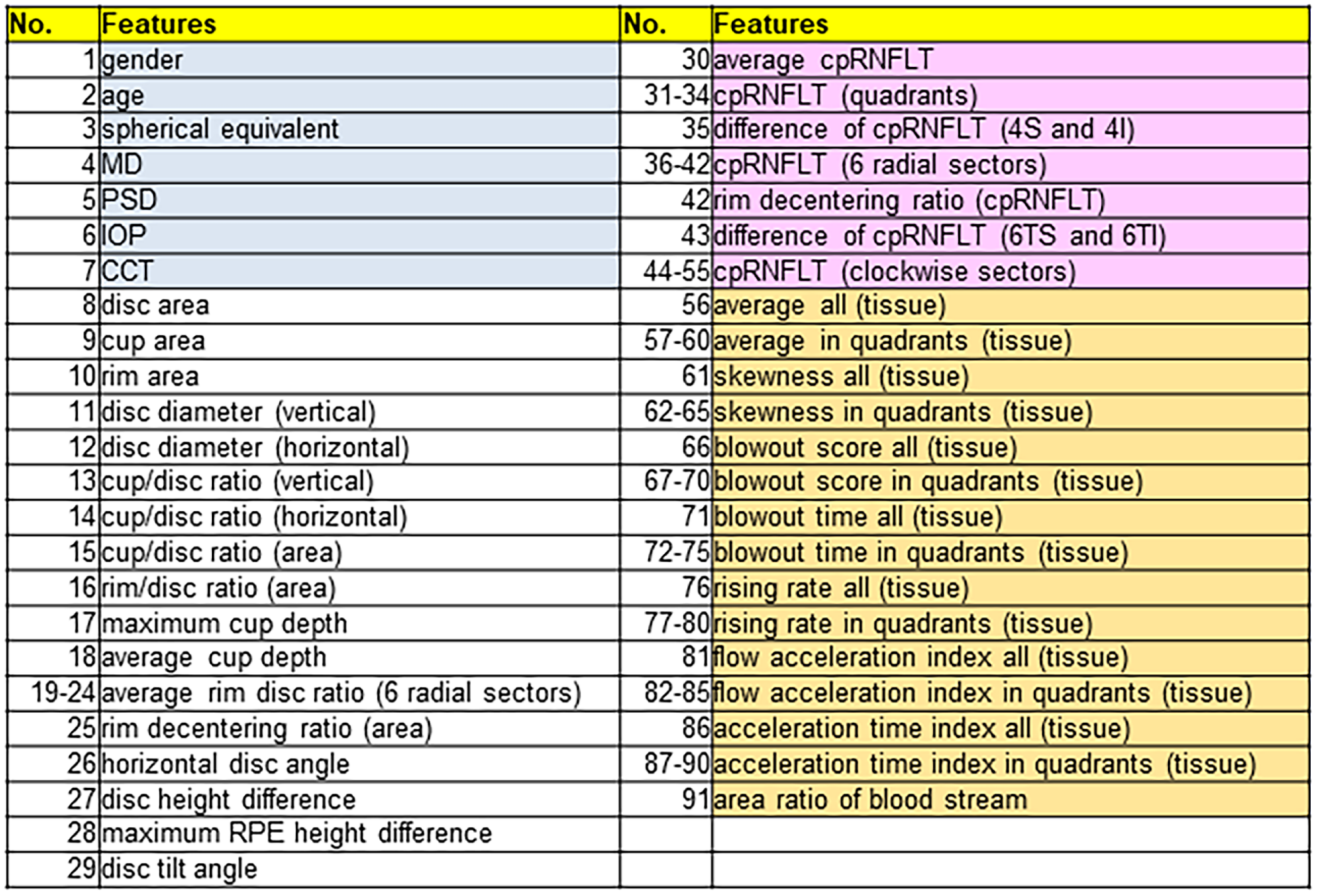
Quantitative ocular parameters from ophthalmic examination instruments. Ninety one types of quantitative data from 7 aspects of patient background (gray column) and 84 types of data, included 22 parameters of optic disc topography (white column), as well as 26 measurement parameters related to cpRNFLT (pink column) and 36 LSFG BF parameters (orange column).

We obtained biographical data for the patients (including sex, age, family history, and medical history) from medical records. All 91 types of data used in this study are listed in Fig 1.

Best-corrected visual acuity (BCVA) was measured with the 5-meter Landolt’s international ring-type chart, and was calculated as the logarithmic minimum angle of resolution (logMAR) with decimal values. Following a slit lamp examination and gonioscopy, IOP was measured with Goldman applanation tonometry. Central corneal thickness (CCT) was measured with anterior-segment OCT (CASIA, Tomey Corp.). Following pupil dilation with 0.4% tropicamide (Mydrin M; Santen Pharmaceutical), OCT parameters, including 22 parameters related to disc topography and 26 parameters related to cpRNFLT, were measured with SS-OCT (DRI OCT Atlantis, Topcon Corp.). CpRNFLT was calculated in the quadrants, 6 radial sectors, and the clockwise sectors. These local measurements were used to quantify asymmetries in cpRNFLT. Mean blood pressure (MBP) and ocular perfusion pressure (OPP) were calculated as follows: MBP = diastolic BP + 1/3 (systolic BP—diastolic BP); OPP = 2/3 MBP—IOP. To assess blood flow (BF) in the ONH, the laser speckle flowgraphy (LSFG-NAVI device, Softcare Co., Ltd., Fukutsu, Japan) was used. Mean blur rate (MBR), an LSFG variable that represents a relative BF index, is expressed in arbitrary units. The accompanying analysis software then automatically divided the region of interest (ROI) into large-vessel and tissue (i.e., capillary) areas and determined specific MBR values in each area (vessel-area MBR: MV; tissue-area MBR: MT). A total of 36 BF waveform parameters were also measured. All data were obtained within a 3-month period.

### Machine learning

To build a machine learning model, we used 91 types of quantitative data from 7 aspects of patient background and 84 types of data, included 22 parameters of optic disc topography, as well as 26 measurement parameters related to cpRNFLT, and 36 LSFG BF parameters (Fig 1). We used a neural network (NN) as the machine-learning classifier, with a structure of 9 input layer units, 8 hidden layer units, and 4 output layer units. After standardization to the training data, we used minimum redundancy maximum relevance to quickly limit the candidate features to 15, and then selected the characteristics with the best classification performance with a genetic algorithm. We used Cohen's Kappa of 10-fold cross validation (CV) for an evaluation index of the hereditary classification performance.

## Results

Demographic data of this study was listed in the Table 1 and assigned data was listed in Table 2.

**Fig 2:**
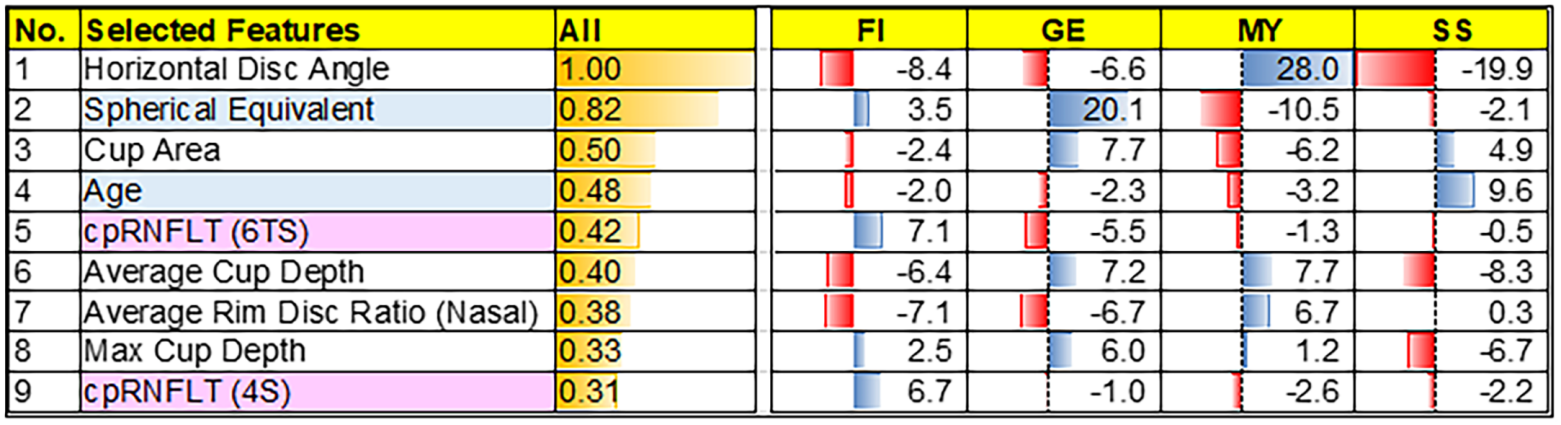
Feature contribution to Nicolela’s classification. The nine most important discriminative characteristics selected by the NN were listed as high contribution order. Overall, horizontal disc angle was the most contributed characteristics of Nicolela’s classification. Contribution to each optic disc type, was also calculated. The value was the relative value of deviation from the mean of each feature. For example, in aspect of age, SS that only has the positive value means SS tends to have older age, compared to other types.

The accuracy and Cohen’s Kappa 10-fold CV were 91.2% and 0.88 for the NN. The nine most important discriminative characteristics selected by the NN were spherical equivalent, age, average rim disc ratio (nasal), average cup depth, horizontal disc angle, 6-sector superior-temporal cpRNFLT, superior-quadrant cpRNFLT, maximum cup depth, and cup area (Fig 2).

Disc-type classification by the NN matched the test data at rates of 66.7% for FI, 93.3% for GE, 83.3% for MY, and 100.0% for SS, and recall at rates of 75.0% for FI, 93.3% for GE, 93.8% for MY, and 80.0% for SS. Overall, the NN had the high validated accuracy against the test data, at 87.8% (Cohen’s Kappa = 0.83).

**Fig 3:**
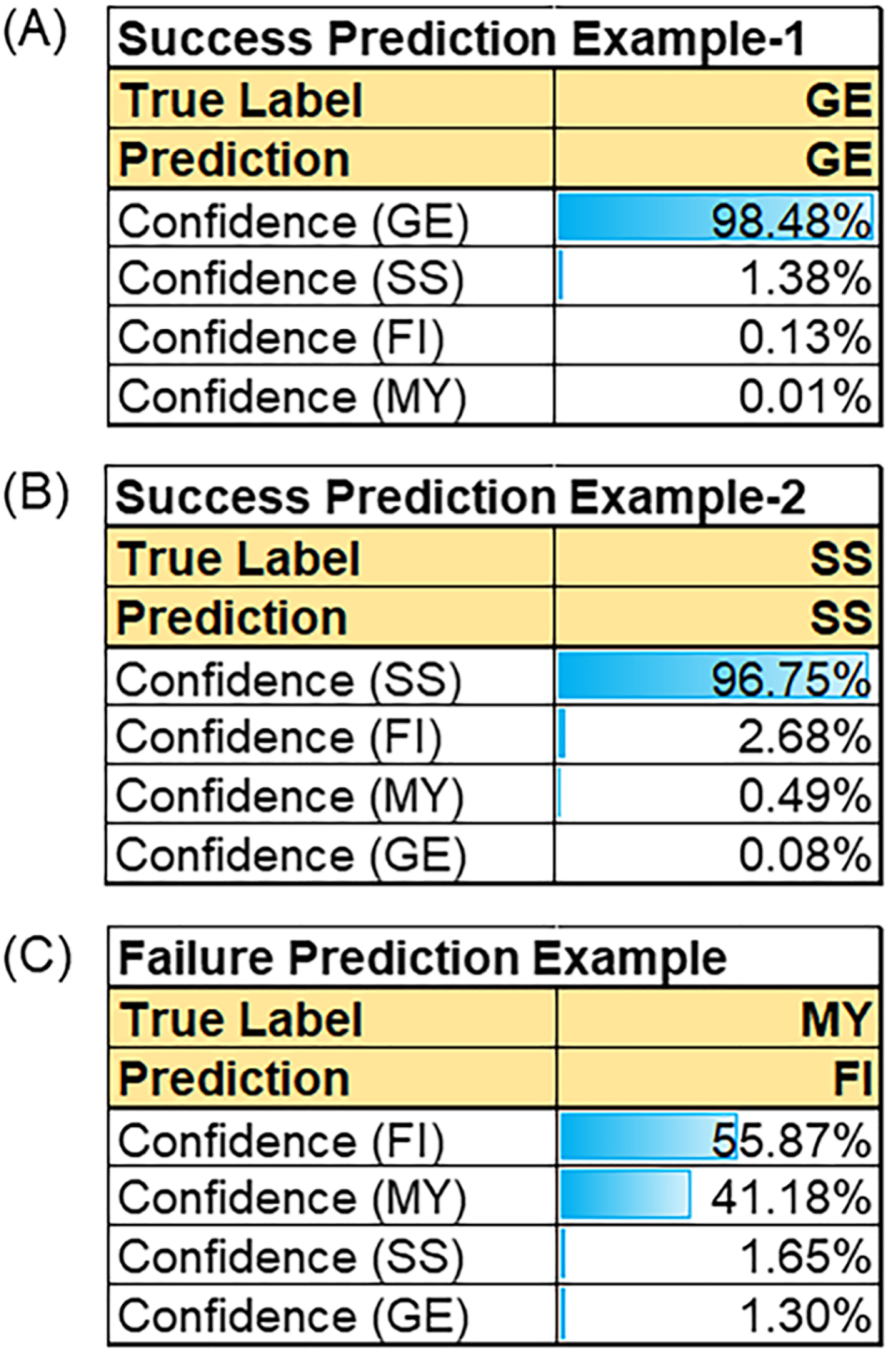
Prediction examples of 3 glaucoma patients. The confidence level of the each Nicolela’s type calculated by the NN model. (a) and (b) showed the NN model predicts accurately with high confidence level as GE (a) and SS (b). (c) NN model predicts with mistake, when discs had a mixed type (FI and MY).

Fig 3 shows the confidence level for the classification of each of Nicolela’s types, as performed by the NN. Fig 3a and 3b show cases with accurate classifications and a high confidence level. Fig 3c shows a case where two potential disc types was assessed by our NN system.

## Discussion

This report describes a new machine-learning algorithm to classify optic disc topography, based on 91 quantitative data types derived from patient background, OCT-measured structure, and blood flow. The NN had the high accuracy, with a match rate to the validated data of 87.8%. Previously, we reported [12] an identification rate of 80.0% and a Cohen's Kappa of 0.73. Thus, the algorithms described in this study promise to help doctors to classify the disc type of patients into Nicolela’s 4 types and to provide useful information for glaucoma care.

Accurate functional classification of glaucoma is an important way of identifying risk factors for progression, which can occur even with successful IOP-lowering treatment. Reported IOP-independent risk factors for NTG progression include disc hemorrhage [4,17], arteriosclerosis [4,18], migraine [19], blood flow abnormalities in the optic nerve (derived from increased resistance in the retrobulbar vessels) [20], Flammer syndrome [5,21], hypotension [22,23], and night dip [24]. Additionally, Nicolela’s 4 types of disc morphology have each been linked to various factors, such as spasm, myopia, IOP, and high or low retrobulbar blood flow. Previously, we also found that the lamina cribrosa was thinner in GE discs, and that lamina cribrosa thickness was independently associated with cup size and tissue blood flow in the optic nerve head [25]. All these findings point to the importance of Nicolela’s disc types for the functional classification of glaucoma. In addition to identifying at-risk patients, classifying glaucoma into progressive subtypes is very important for analyzing large patient data sets. Our algorithm is a first step towards the establishment of machine-learning methods for the functional classification of glaucoma, and in the future, we hope that such methods will open new directions for research and improve treatment outcomes, not only for established IOP-lowering approaches, but also for newly reported IOP-independent strategies, such as those targeting blood flow and oxidative stress.

It is widely believed that glaucoma diagnosis would be subject to less variation if the relationship between structure and function were better understood. Supervised learning approaches have been most frequently used to discriminate between glaucomatous and non-glaucomatous eyes, and most published studies in the field of glaucoma research have used supervised machine learning techniques to improve diagnoses [14]. Belghith et al. showed that the AUC to differentiate glaucoma was 0.91 for Bayes, 0.69 for an artificial NN, and 0.6 for a support vector machine (SVM) [13]. Mookiah et al. compared different methods of machine learning and found that a simple linear SVM was superior to decision tree, nearest neighbor, naïve Bayes, and probabilistic neural network (PNN) analyses for the diagnosis of age-related macular degeneration [15]. Torok et al. investigated six machine-learning algorithms, including an SVM, recursive partitioning, random forest, Naïve Bayes, logistic regression, and K-nearest neighbor (k-NN), and found that the results of recursive partitioning could most accurately screen for diabetic retinopathy [26]. Therefore, the accuracy of machine-learning algorithms varies with different conditions.

Here, we found that 9 clinical and morphological characteristics were important components for automatically classifying optic disc type, including spherical equivalent, age, average rim disc ratio (nasal), average cup depth, horizontal disc angle, 6-sector superior-temporal cpRNFLT, superior-quadrant cpRNFLT, maximum cup depth and cup area. Our previous research [12] showed that six parameters were most significant for disc type discrimination: disc angle (horizontal), average cup depth, cup/disc ratio, rim-decentering ratio, average rim/disc ratio (upper and lower nasal). These previous findings are closely consistent with the present study. Generally, eyes with the MY disc type have a low spherical equivalent, and the onset of glaucoma occurs at a younger age. Moreover, MY discs are tilted temporally, resulting in a high horizontal disc angle, and have a high nasal cup to disc area ratio. On the other hand, GE discs generally have a thin nasal rim and a large average and maximum cup depth and cup area, while SS discs have shallow cupping and are associated with the onset of glaucoma at an older age. FI discs showed thickening of the cpRNFLT in the 6-sector superior-temporal and superior quadrants. These characteristics of different disc types likely underlie the similarities between the most important parameters for discriminating disc type in each machine-learning model. Furthermore, we calculated the contribution of these characteristics to Nicolela’s types (Fig 2), and found that in general, horizontal disc angle was the most useful for automatic classification. All these characteristics will be recognizable to experienced clinicians, and are likely similar to their own sense of the most important contributors to disc classification. Interestingly, however, the NN in this study selected two quantified parameters from patient background information and seven from purely digital, OCT data, without any parameters derived from LSFG. This may be because the definitions of Nicolela’s 4 disc types do not include any blood flow parameters. Thus, future research into functional OAG classification based on LSFG parameters may be necessary. Nevertheless, this study showed that it may be possible to use OCT data to classify the disc into Nicolela’s 4 types [6], without reference to blood flow parameters.

The NN model allowed calculation of the confidence level of the prediction (Fig 3). This showed that many individual predictions had high confidence levels (Fig 3a and 3b), and that the overall accuracy of our method was 87.8%. We returned to our data to reconsider why the NN misidentified certain discs, and found that in most cases, the correct disc type had been the second choice. When we recalculated the accuracy of the model to include the first and second choices, the accuracy increased to 95.9%. This result may reflect the real-life experiences of glaucoma specialists, who are sometimes unsure which disc type is correct, and classify discs as having a mixed type, such as mixed FI and MY discs (Fig 3c). Thus, the presence of mixed disc type influences the accuracy of disc type classification with our machine-learning system. Nevertheless, each disc type had a good confidence level, including more than 80% for the pure MY disc type, which should help future efforts to find accurate classification methods and help improve daily clinical glaucoma care.

Limitations of this study included a cross-sectional design, the inclusion of only Japanese subjects, and a relatively small study population, which may have affected the statistical power of our analyses. However, the entire analysis was performed 3 times, with randomized selection for the training and validation groups, which supports the accuracy of our classification system. An additional limitation was the unavoidable inclusion of factors that might have influenced the OCT and LSFG measurements, such as myopia. In Asia, the combination of myopia and glaucoma is very common. Nevertheless, we excluded cases with high myopia and adjusted the number of cases with glaucomatous myopic disc to minimize possible bias.

In conclusion, this study used various parameters derived from OCT and LSFG and from biographical and clinical data to set up an automated, objective, machine-learning based method for the classification of Nicolela’s 4 disc types, with a success rate of 87.8%. This technology has the potential to standardize diagnostic criteria for optic disc classification in future clinical trials, thereby reducing possible bias. However, in daily practice, this technology is not superior to the judgements of experienced glaucoma clinicians. Thus, the techniques described here have the potential to be very useful, powerful tools for supporting clinical decision making. Our findings should help improve the classification of OAG and lead to better glaucoma management, by demonstrating the confidence level of predictions for each disc type.

## Supporting information

S1 File Demographic data and quantitative ocular parameters from ophthalmic examination instruments. F1 File contains 91 types of quantitative data from 7 aspects of patient background and 84 types of data, included 22 parameters of optic disc topography, as well as 26 measurement parameters related to circumpapillary retinal nerve fiber layer thickness from OCT, and 36 blood flow parameters from laser speckle flowgraphy.
(CSV)
